# Histo- and cytopathological findings in the brain of two fire fatalities

**DOI:** 10.1007/s12024-025-01018-3

**Published:** 2025-04-25

**Authors:** Simone Bohnert, Benjamin Ondruschka, Helmut Heinsen, Michael Bohnert

**Affiliations:** 1 Institute of Forensic Medicine, University of Wuerzburg, Versbacher Str. 3, 97078 Wuerzburg, Germany; 2 Institute of Legal Medicine, University Medical Center Hamburg-Eppendorf, Butenfeld 34, 22529 Hamburg, Germany

**Keywords:** Forensic neuropathology, Cytoarchitectonics, Gallocyanin, Nissl staining, Burn, Brain

## Abstract

There are only few reports on the neuropathologic findings of fire victims. We investigated brain tissues of a 44-year-old and a 77-year-old man for neuropathologic examination with dehydration, embedding in celloidin, sectioning at 400 μm, and staining with gallocyanin. Microscopically, neurons were less well stained than those from an 87-year-old woman who died of cancer and whose brain had been fixed in formalin for three months. Glial cells were optimally stained. We observed local, laminar and disease-related qualitative and quantitative differences in the amygdaloid complex, temporal allo- and isocortex together with hyperchromatic staining of the medullary layer in the temporal lobe of both fire cases. The vasculature was well preserved and free of blood cells or clotted blood. The heat in fire deaths apparently acts as a kind of fixation, similar to the intention of formalin use, without the confounding effects of agonal and postmortem factors. Heat is most likely a major factor in microwave fixation. Thick gallocyanin-stained sections allow intuitive visual diagnosis of local and laminar neuronal degeneration or gliosis and have the potential to enhance and refine neuropathology-related diagnoses.

## Macroscopic findings

### Case 1

The corpse of a 44-year-old man was discovered in a garden shed following an explosion that resulted in a fire. The deceased had previously exhibited symptoms of a mental disorder accompanied by suicidal ideation and behavior. Four days after the external postmortem examination, an autopsy was conducted on the deceased, who was 176 cm in height and weighed 72 kg. The external examination revealed that the body had sustained fourth-degree burns covering 100% of the surface area and that the right foot had been amputated as a result of the fire. The presence of soot deposits was observed in the upper and middle airways, and the mucous membranes of the trachea and bronchi exhibited evidence of damage caused by heat. The concentration of carboxyhemoglobin (CO-Hb) in the corpse’s blood was 8%. There were no intracranial hemorrhages. Overall, the autopsy revealed no signs of mechanical trauma or pathological changes in the internal organs. The cause of death was assumed to be heat inhalation trauma.

### Case 2

The body of a 77-year-old man was discovered at the base of the staircase of a residential building that had been extensively damaged by fire following an explosion. It was reported that he had been experiencing mental instability for an extended period. A postmortem examination was conducted six days after the decedent’s death. The deceased was 165 cm tall and weighed 75 kg. As in the previous case, the body displayed a predominantly fourth-degree burns pattern across 100% of the body surface area. Similarly, the assumption was that heat inhalation trauma was the cause of death, given the presence of soot deposits and heat-related mucosal detachment in the larynx, trachea, and main bronchi. The concentration of carboxyhemoglobin (CO-Hb) in the corpse’s blood was 22%. Once more, there was no evidence of mechanical trauma and no evidence of bleeding within the skull.

After opening of the skulls, there was no evidence of mechanical trauma and intracranial bleeding. Both brains were characterized by a consistency similar to formalin immersion-fixed brains after extended fixation of 2 to 3 months. After removal of the brains from the skull the brains did not deform when placed onto the autopsy table. The brain of case 1 weighed 1530 g, that of case 2 1308 g including arachnoidea and cerebral vasculature. The cerebral sulci of case 2 were wider and the arachnoidea was fibrotic compared with the brain of case 1. The brains were dissected into coronal 15 mm thick serial slices. The cut surface exhibited no focal alterations, such as hemorrhages, softening, or suspected neoplastic regions. One slice from each case at the level of the mammillary body was placed into diluted formalin (1 part 37% formalin diluted with 9 parts of tap water) for 4 weeks, dehydrated with ethanol, embedded into celloidin, serially cut into slices of 400 μm thickness, stained with gallocyanin and coverslipped like reported before [1].

## Microscopic findings of stained hemispheric slices

**Fig. 1:**
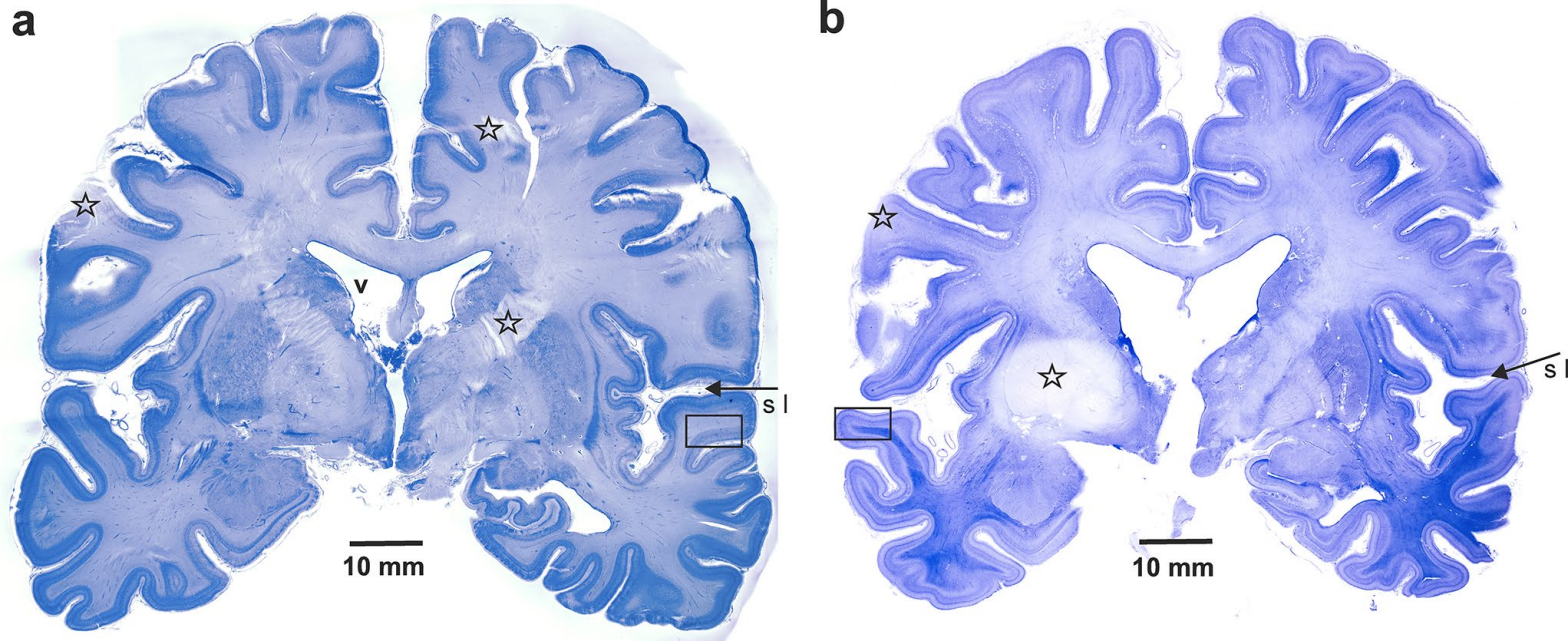
Overview of gallocyanin-stained 400 μm thick frontoparallel brain slices at the level of the mammillary bodies. (**a**) *Case 1* 44-year-old man, (**b**) *Case 2* 77- year-old man. The asterisks indicate cutting artefacts of unfixed brain tissue

Gallocyanin is a progressive Nissl stain that has been demonstrated to bind to nucleic acids [2]. It is well established that neurons are known to contain high concentrations of DNA and RNA. The cerebral cortex and subcortical nuclei are distinguished by their high neuron density. The delineation of regional variations in neuron density and arrangement is facilitated by using 400-µm-thick sections (Fig. 1a, b). The telencephalic medullary layer is primarily comprised of oligodendro- and astrocytes, which are virtually devoid of Nissl substance in light microscopic preparations. This results in a faint staining of the telencephalic white matter. However, upon closer examination, the periventricular parts of the central white matter appear lighter, whereas the medullary rays in the gyri appear darker. This phenomenon is particularly evident in the temporal lobe, which is demarcated from the frontal lobe by the lateral sulcus (S l).

It is imperative to acknowledge that the process of dissecting unfixed brain tissue is susceptible to various forms of tissue deformation, loss, and uneven section thickness. The asterisks in Fig. 1 denote staining differences attributable to artefactual variations in gross section thickness and tissue loss.

Both slices were selected from comparable planes of coronal sections. However, a discernible variation in total size is evident, with the gyri of the 77-year-old male subject exhibiting a reduction in size, accompanied by an expansion of the sulci and the ventricular system (v). A stereologic estimation employing point-counting methods [3] yielded a total hemispheric area of 55.8 cm² vs. 45.5 cm² (-18.6%), 21.2 cm² vs. 17 cm² (-19.9%) of cortical tissue, and 34.6 cm² vs. 28.4 cm² (-17.9%) of subcortical tissue, which encompasses the white matter and subcortical nuclei. The data in brackets quantify the amount of nervous tissue deficit in the 77-year-old male subject compared with the 44-year-old male subject.

**Fig. 2:**
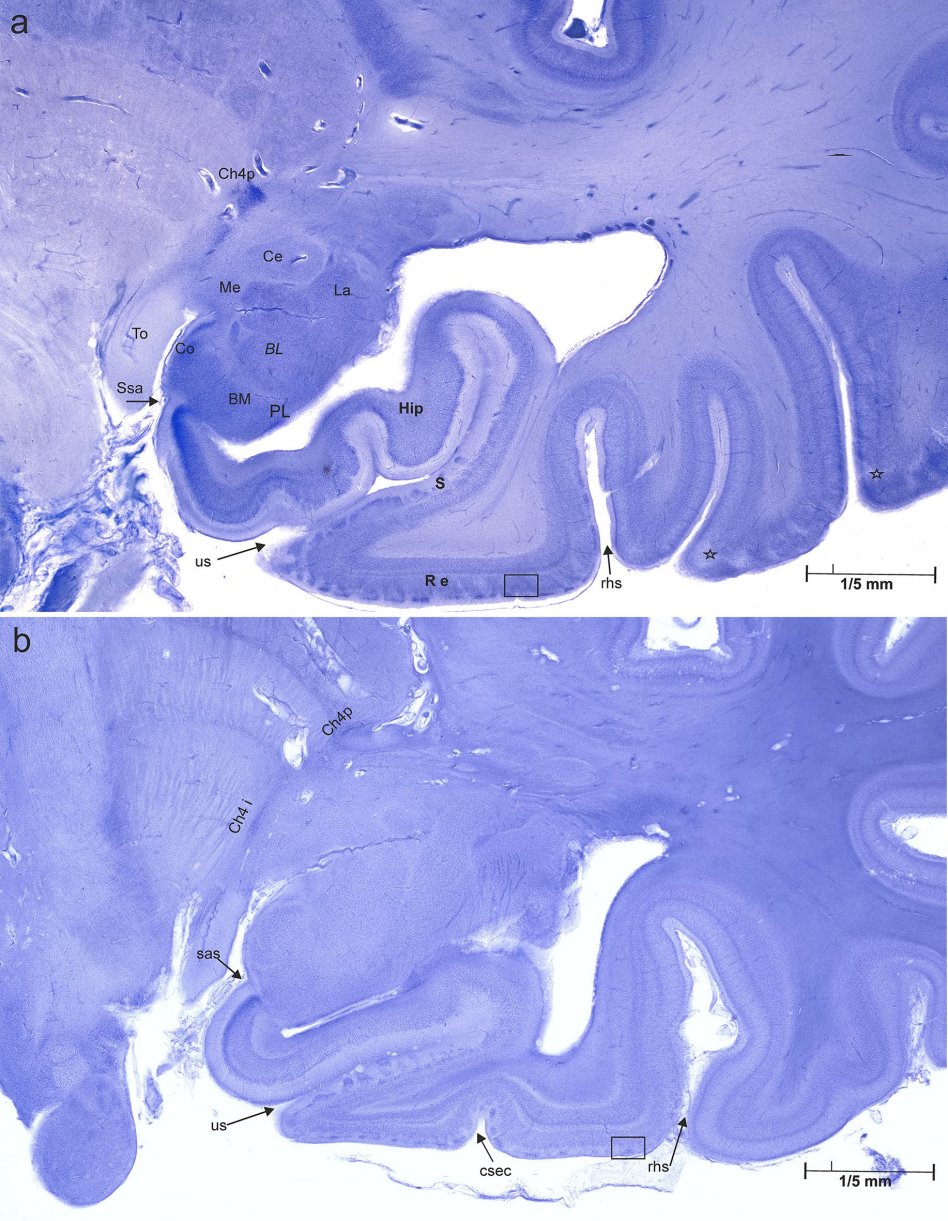
Close-up view of the right mediobasal temporal lobe. (**a**) Case 1, 44-year-old man, (**b**) Case 2, 77- year-old man

The medial temporal lobe comprises corticoid, allo-, periallo-, and isocortical elements. The optic tract (To) courses in close proximity to the amygdaloid complex [4]. The shallow semi-annular sulcus demarcates the boundary between the amygdaloid cortical nucleus (Co) and the anterior hippocampal fields (Fig. 2a).

Individual nuclei of the amygdaloid complex are grouped [5]. The central nucleus (Ce) and the medial nucleus (Me) form the deep centromedial group. They are located in close proximity to the cholinergic and chromophilic nuclear complex of Mesulam’s Ch4p [5], a component of the basal forebrain complex or Meynert’s basal nucleus [6]. In Fig. 2b Ch4i, an additional nuclear group of the basal forebrain complex, can be identified. The superficial cortical amygdaloid region is divided into several sectors. In Fig. 2a, only the cortical nucleus (Co) and its subdivisions could be traced. The basolateral nuclear group consists of the lateral (La), basolateral (BL), basomedial (BM), and paralaminar (PL) amygdaloid nuclei. Nuclear groups and individual amygdaloid nuclei are characterized by specific afferent and efferent connections and functions [7].

The three-layered anterior hippocampal fields (Hip), together with the subicular fields (S) and the entorhinal cortex (R e), constitute the hippocampal formation [8], which extends from the semiannular sulcus (S sa) to the rhinal sulcus (S rh). The regions of the hippocampal formation are covered by an allocortex. The allocortex has fewer layers than the six-layered isocortex. A closer look at the entorhinal cortex seems to contradict this rule, as Braak identified up to 12 entorhinal layers [9, 10]. However, ontogenetic studies show that these sublayers arise from three-layered precursors [11]. Large chromophilic stellate or pre-alpha cells are specific cells of the entorhinal region. They are arranged in clusters and constitute the second layer of the entorhinal cortex. As a rule, the pre-alpha clusters are replaced by a chromophilic layer II of small cells in the medial wall of the rhinal sulcus. This feature marks the transentorhinal region, a region of transition between allo-and isocortex.

Figure 2b differs from Fig. 2a by a general decrease in staining intensity or pallor. This is evident when comparing Ch4p, the chromophilic neurons in the amygdaloid nuclei, and the entorhinal pre-alpha cells. In addition, Ce, Me, BM, and BL show sectors with significant neuronal loss along with thinning of layers in the hippocampal formation.

**Fig. 3:**
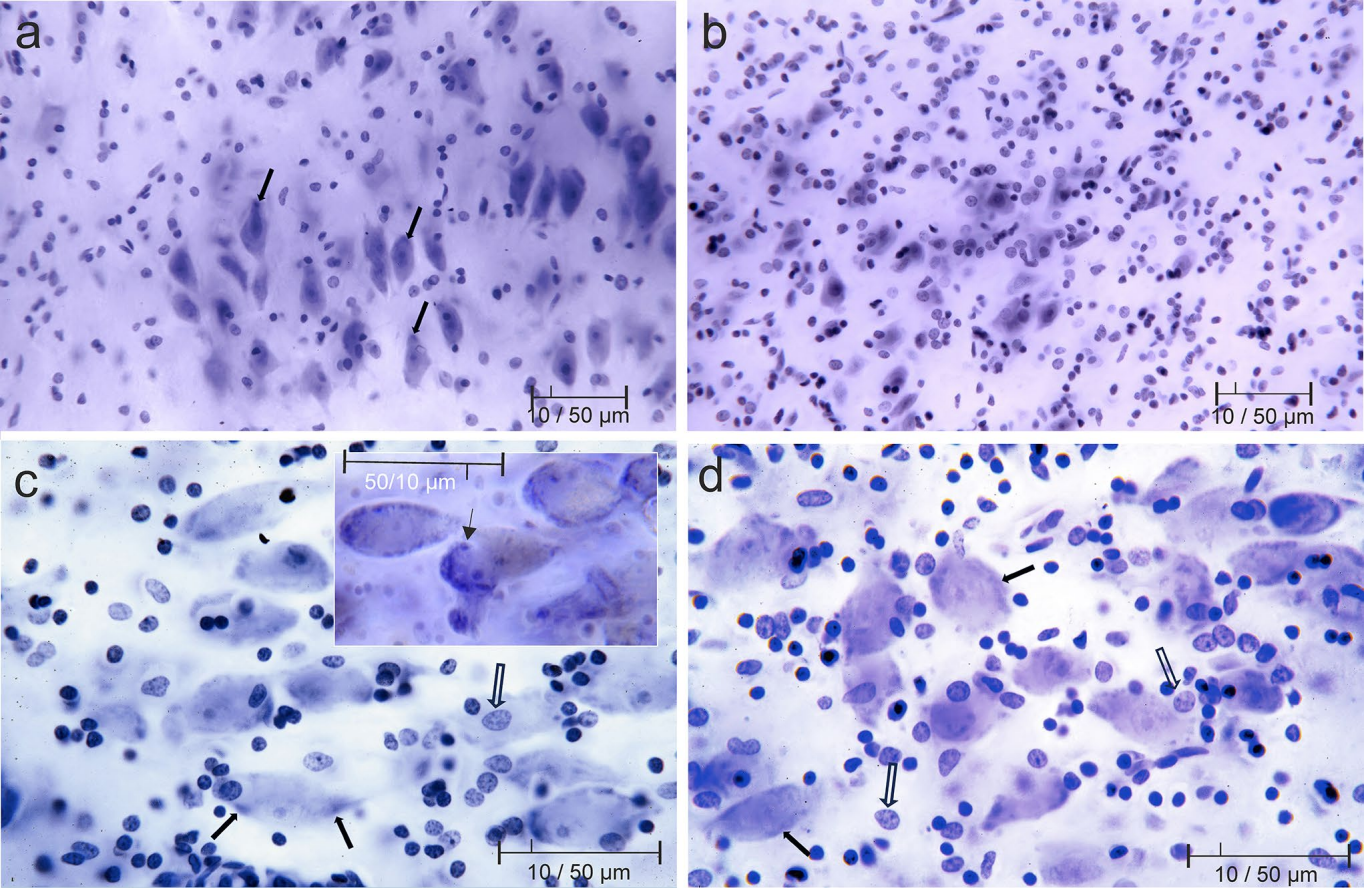
**a**) and **b**) entorhinal pre-alpha cells, **c**) and **d)** large projection neurons of Meynert’s basal nucleus Ch4 p. **a** and **c** from Case 1, **b** and **d** from Case 2. *Inset***c** 87-year-old woman, control case (vaginal cancer as cause of death)

The two types of neurons are distinguished by their size, shape, and radiating bi-multipolar dendritic processes. Nissl bodies accumulate at the base of the dendritic arbor of pre-alpha cells (Fig. 3a, arrows) and the soma of large Ch4p cells (Fig. 3c and d arrows). A further distinguishing feature of pre-alpha cells is the greater chromaticity of their perikaryon compared to that of large Ch4p cells. The presence of nucleoli was observed in both cell types, while the nuclear membrane was only barely discernible. We have inserted the *inset* in Fig. 3c to visualize the amount and distribution of Nissl bodies in 87-year-old female control case conventionally fixed with formalin. The arrow in the inset of Fig. 3c points to a lobated nucleus with two nucleoli. The central parts of large basal Meynert neurons are filled with a light yellow to brown material, lipofuscin, which is known to increase with age and to dislocate Nissl bodies to peripheral perikaryal (cytoplasmatic) compartments of the nucleus basalis Meynert neurons (Fig. 3d arrows). After formalin fixation Nissl bodies are more numerous, their variation in size and distribution can be better assessed after formalin fixation compared to brain tissue subjected to heat exposure (Fig. 3c and d).

The nuclear staining of glial cells contrasts with that of neurons. The nuclear membrane and heterochromatin are clearly delineated (Fig. 3c and d blockface arrows). Astrocytes are easily diagnosed; the differential diagnosis of oligodendrocytes and microglial cells is uncertain.

The neuron density of pre-alpha cells in the entorhinal region of the 77-year-old male subject is lower compared with the identical region in the 44-year-old male subject. Conversely, the glial cell density exhibited a conspicuous enhancement in the older individual (Fig. 3a and b).

Cytoarchitectonic studies require low-power magnification and strictly perpendicular sections through defined regions of the gyri under study (crown, wall, fundus). Cytoarchitectonic features of isocortical fields are intermediate between agranular (motor) and granular (koniocortical) sensory fields. They consist of 6 layers designated by Roman numbers, beginning with the superficial layer I and ending with the deep layer VI. Layers III, V, and VI are subdivided into sublayers termed a, b, and c [12].

The size, shape, orientation, and density of neurons in the cortical layers, as well as their relative thickness, provide criteria for delineating cortical fields. Layers I through VI are stacked horizontally, however, with higher magnification fibers are seen to leave the central medullary ray of the gyri and arch vertically through the cortical layers. These radial fibers cause a region-specific cortical striation and vertical columnar organization of neurons. The number and thickness of radial fibers provide additional criteria for delineating architectural fields. Rectangles in Fig. 1a and b illustrate the topography in the whole slice.

**Fig. 4:**
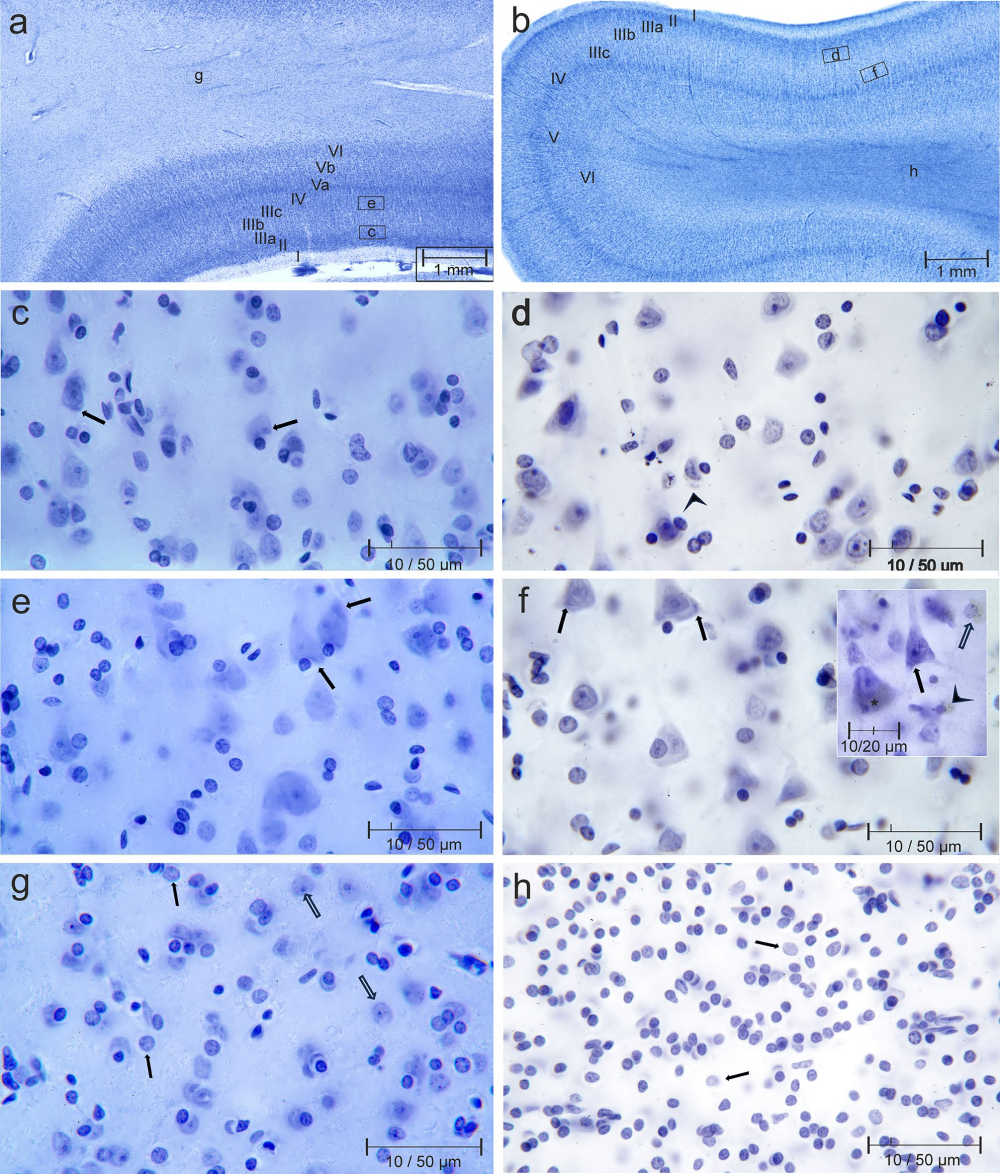
**a**) and **c**) superior temporal gyri at low power magnification; rectangles and letters indicate the location of figures **c-h** taken with an oil immersion microscopic lens; **a**, **c**, **e**, **g** Case 1, 44-year-old man; **b**, **d**, **f**, **h** Case 2, 77- year-old man. *Inset*** f** 87-year-old woman, control case (vaginal cancer as cause of death)

Low-power magnification reveals significant differences between the two cases under investigation, including pallor of the supragranular layers III and loss of layer IV granule cells. The presence of intensely stained layer Va pyramidal cells and neuron loss in layers Vb and VI create a rudimentary horizontal layering, which is already recognizable in the overview panel (Figs. 1b and 4b). The remaining radial fibers appear less numerous and thicker.

Another striking finding is the markedly increased chromophilia of the central medullary ray in the 77-year-old male subject. The alterations in stainability are not confined to the central medullary ray; they are also present in the central gray matter (Fig. 1b).

Pyramidal cells constitute approximately 80% of all telencephalic cortical neurons [13]. The rectangles in Fig. 4a and b demarcate the position of neurons and glial cells. The staining intensity of neurons, glial cells, and neuropil (diffusely stained background consisting mainly of abundant fine invisible neurites and glial processes) is higher in the 44-year-old male subject. Nissl bodies (or better substance) are rare in IIIa pyramidal cells (arrows in Fig. 4c) and in IIIc pyramidal cells (arrow in Fig. 4e).

The perikaryon of IIIa and IIIc-pyramidal cells is pale in area 22 of the 77-year-old male subject. Only one Nissl body could be identified in a IIIc pyramidal cell (arrow in Fig. 4f). Prominent nuclei and nucleoli contrast with the pale surrounding perikaryon.

The inset in Fig. 4f shows a Nissl body and pyramidal cell dendrites (arrow), lipofuscin granules in the basal parts of a large IIIc pyramidal cell (Fig. 4f, inset, star), fine lipofuscin granules surrounding a reactive astrocyte (Fig. 4f, inset, block arrow) and yellow material engulfed by a microglial cell (Fig. 4f, inset, arrowhead).

The central medullary ray of human telencephalic gyri contains few, if any, neurons in its central parts (Fig. 4g block arrows). The nuclei of oligodendrocytes and far fewer nuclei of astrocytes (Fig. 4, g and h, black arrows) predominate in this part of the gyri. The density of oligodendrocytes is low in the 44-year-old male and very high in the 77-year-old male. The nuclei of oligodendrocytes can be arranged in long rows or in a semicircular fashion surrounding optically bright spaces with a single central astrocyte (Fig. 4h, black arrows).

The coronal plane of the slices of our younger case passes through anterolateral parts of the anterior commissure (C a). The putamen (Put) is still connected to the caudate nucleus (Caud) by the ventral striatum. The anterior perforate substance ventral to Mesulams Ch4i forms the telencephalic floor in this region. It is perforated by the small anterolateral arteries arising from the M1 segment of the medial cerebral artery. These branches supply major parts of the external or internal pallidal globe (Gpe, Gpi), the putamen, parts of the internal capsule (C i) and the head of the caudate nucleus. The origin and course of the perforant arteries are subject to individual variation with different clinical importances [14].

**Fig. 5:**
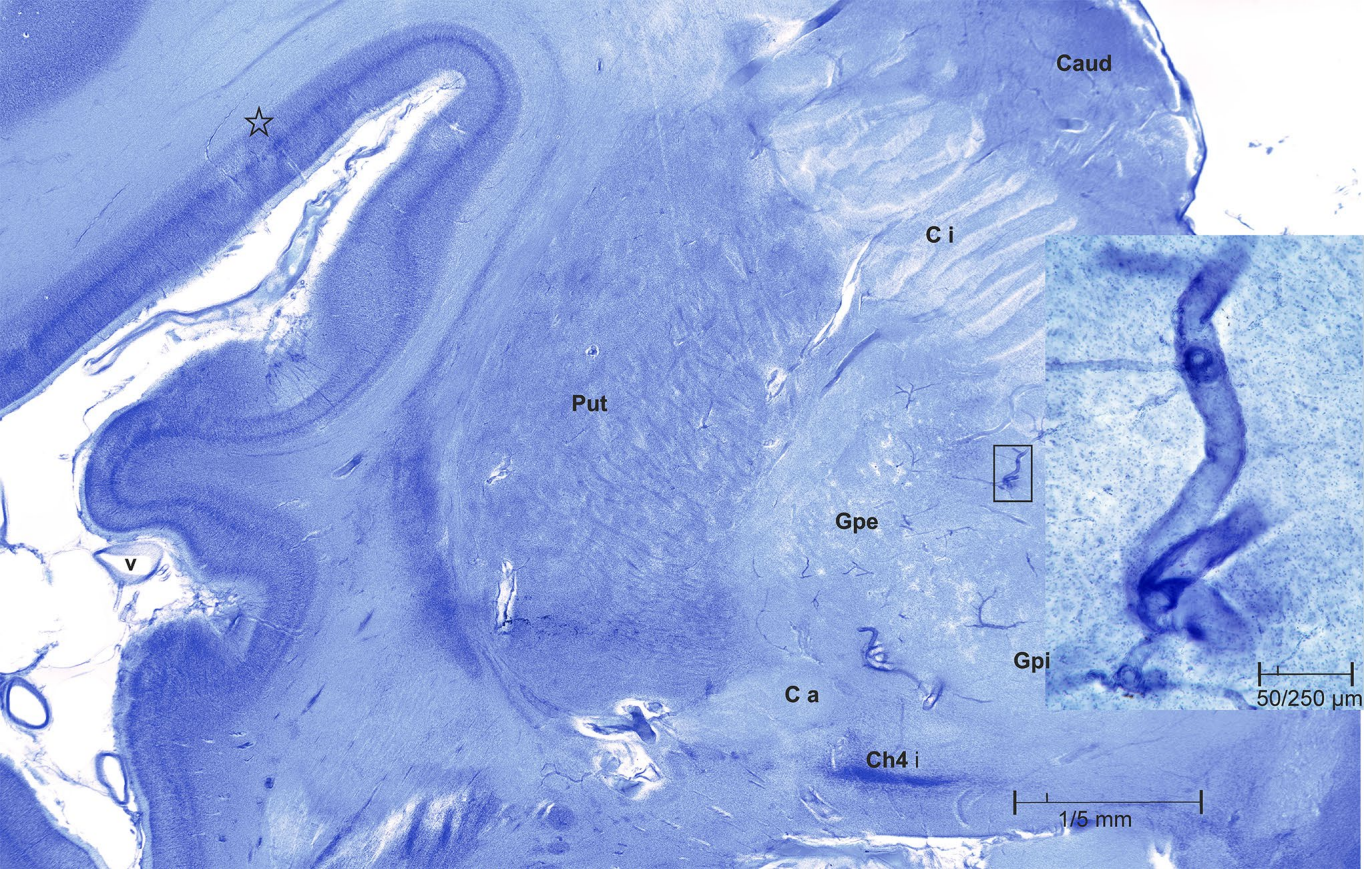
Close-up view of the left basal ganglia and anterior perforate substance of Case 1, 44-year-old man; the inset shows stacked optical slices of two small crossing arteries in the internal pallidal globe

The course of these branches is easy to follow in our thick sections because of their relatively wide lumina. The rectangle at the medial border of the pallidum with the lateral parts of the internal capsule encloses two segments of these arteries. We made optical sections at 40x microscopic magnification and combined the individual optical sections into a coherent 3D stack (*inset* in Fig. 5). The right-angled emergence of the arterioles is noteworthy because it exerts a lifelong hemodynamic stress on the arterial walls at these sites.

## Discussion

While reports on the lungs, airways and skeleton of fire victims are comparatively frequent, there are only very few publications in the forensic literature dealing with brain findings in fire deaths. The most comprehensive paper on this subject was published by Dotzauer and Jacob seven decades ago [15]. Dotzauer summarized previous observations and added detailed findings on additional three cases.

His histologic techniques of formol fixation and celloidin embedding were similar to the one used here. However, he used kresyl violet as a Nissl stain. Unlike gallocyanin staining, the former is a regressive stain. Excess kresyl violet must be washed out under subjective visual control, whereas gallocyanin binds selectively to nucleic acids [16]. In addition, this property prevents co- and overstaining of tissue elements other than nucleic acids and can be applied to extended sections up to 1 mm thickness.

Our results after gallocyanin staining were *similar* in major aspects to those of Dotzauer. As Dotzauer reported Nucleoli, nuclei, and the histologic equivalent of rough endoplasmic reticulum, Nissl bodies, were stained. In addition, regional, cell type-specific, and age- and disease-related differences in staining intensity were observed. In general, the staining intensity of neurons in our two fire victims was less intense than in a conventionally formalin-fixed whole brain of an 87-year-old woman (death from extensive vaginal cancer), and only short stumps of pyramidal cell dendrites could be traced. In addition, the yellow tint of lipofuscin was lost in cases of fatal burns (Fig. 4f inset arrowhead and block arrow versus Fig. 4d arrowhead). Cells appeared swollen, the base of pyramidal cells was rounded, whereas neuronal perikarya fixed exclusively in formalin were shrunken and triangular. On the other hand, nuclear heterochromatin of glial elements was clearly visible in contrast to glial nuclei after fixation with unbuffered formalin. This is consistent with Dotzauer’s findings.

*Contrary* to Dotzauer’s observation, we did not find any artifactual changes in the orientation and shape of vascular and perivascular cells, and no enlarged Virchow-Robin perivascular spaces. The lumina of arteries and veins in our two cases were unusually wide and completely empty. Only the walls of the periventricular veins were sometimes diffusely stained with gallocyanin. The state of the vasculature reminded us on successful perfusion fixation in experimental animal and human brains with acute death and only short postmortem intervals.

Neuropsychiatric symptoms were reported as pre-existing medical condition in both cases. We diagnosed neuronal loss in the cortical and basomedial nuclear groups of the amygdala, laminar neuronal loss with gliosis in the entorhinal cortex, and laminar neuronal loss in the supra- and infragranular layers in the temporal isocortex of the 77-year-old man. This neuronal loss is consistent with the seminal Braak and Braak staging [17] of the temporal and topographic appearance and spread of argentophil Alzheimer fibrils. They spread in a retrograde fashion from allo- to isocortical regions and ultimately cause irreversible neuronal loss in late stages. In addition, the chromophilia of the medullary layer in the cortical gyri and the close spacing together with the high density of oligodendrocytes and the decrease in the number of fine radial fibers are probably the consequence of anterograde or retrograde axonal degeneration of dying cortical association and projection neurons in layers IIIc and V. Widespread neuronal loss in cortical layers seems to be the morphological correlate of dementia in a number of neurodegenerative diseases [18]. Immunohistochemical (IHC) staining with antibodies against hyperphosphorylated tau, aß-protein, and alpha-synuclein would confirm the diagnosis.

The neuropathological basis of the suicidal ideation and behavior of the 44-year-old man remains unclear. Laminar focal pallor could be seen in his right inferior temporal gyrus (Fig. 2a, asterisks) and the left inferior frontal gyrus (Fig. 5, asterisks). The role of the pallidal status cribrosus on mental health in our case is enigmatic. However, these lesions are small and localized. They could be the result of previous local vascular spasms or simply artifacts.

We were able to demonstrate that macroscopically intact brains after fatal fire accidents, mounted in celloidin and sectioned at high thickness, increase the diagnostic tools for neuropathologic microscopic examination of sliced or whole human brains. In our experience, after a short training period, gallocyanin-stained thick sections allow intuitive visual diagnosis of local and laminar neuronal degeneration or gliosis starting at a lower limit of deficit of about 30%. Laminar neuronal loss in the entorhinal cortex and interconnected hippocampal fields may explain early short-term memory deficits in Alzheimer’s disease [19]. The nuclei of the amygdaloid complex are interconnected with the prefrontal cortex, the sensory association cortex, intrinsically, and with brainstem and spinal cord nuclei. Neurons in the amygdaloid complex are subject to tau- and alpha-synuclein-positive inclusions in Alzheimer’s disease and Parkinson’s disease, respectively, and are thought to cause non-motor symptoms in early Parkinson’s disease [7, 20]. Recent functional magnetic resonance observations have also been interpreted as suggesting an important role for the amygdaloid complex in schizophrenic symptomatology [19].

Already Dotzauer discussed the impact of temperature, location, extent, and duration of fire on preservation of the human central nervous system in the skull. Marani and Boon [20] and Giberson and Demaree [21] emphasized the importance of controlled heat flow and the addition of glutaraldehyde and osmium tetroxide for optimal fixation of tissue for light and electron microscopic examination.

Given that this type of heat-fixed tissue could also react with diagnostic antibodies for IHC, it would greatly enhance the scientific tools available to forensic pathologists and appears to be especially practical in countries where neuropathological examination is highly regulated or where standard fixation may not be available. It could serve as a valuable research tool to study brain tissue of normal and diseased humans with neurologic or psychiatric diseases *without* relevant agony and *rapid* postmortem autolysis.

In addition to the comprehensive evaluation of neural and glial integrity, it is imperative to corroborate and expand our diagnoses through the implementation of supplementary microscopic and IHC methodologies. A number of strategies could be employed in this regard. A potential approach involves the extraction of specific segments from parallel unstained 400 μm thick sections, followed by their embedding in paraffin, cutting at a thickness of 5 μm, and subjecting them to IHC [1]. Alternatively, in place of unbuffered formalin fixation, different mixtures of glutaraldehyde and buffers could be tested on 1.5 cm thick sections. Finally, defined tissue segments from the sliced brain could be cut out, immersed in liquid nitrogen, cut on a freezing microtome, and processed with IHC.

We would also like to point out that further studies are necessary to verify this observation. With only two fire cases and one control case, the comparability of specific constellations is limited and should be addressed in subsequent investigations in the form of case-control studies; however, thick gallocyanin-stained sections have the potential to enhance and refine neuropathology-related diagnoses.

## Data Availability

N/A.
